# A Prospective Evaluation of Health Literacy Levels of Pregnant Women in Antenatal Classes: Impact on Delivery Outcomes in Nulliparous and Multiparous Women

**DOI:** 10.3390/diagnostics14222580

**Published:** 2024-11-17

**Authors:** Sakine Rahimli Ocakoglu, Zeliha Atak, Ozlem Ozgun Uyaniklar, Gokhan Ocakoglu

**Affiliations:** 1 Department of Obstetrics and Gynecology, Bursa City Hospital, Nilufer 16110, Bursa, Turkey; zeliha.atak@hotmail.com (Z.A.); ozlemuyaniklar@gmail.com (O.O.U.); 2 Department of Biostatistics, Bursa Uludag University Faculty of Medicine, Nilufer 16059, Bursa, Turkey; gocakoglu@gmail.com

**Keywords:** health literacy, antenatal classes, primary cesarean section, nulliparous and multiparous women, delivery outcomes

## Abstract

**Backgrounds/Objectives**: Modern technology and educational activities, such as antenatal classes (ACs), increase patient informedness in medicine and improve collaboration between physicians and patients. The present study aimed to evaluate and compare the impact of maternal health literacy (HL) on delivery outcomes between nulliparous and multiparous women who attended antenatal classes. **Methods**: This prospective study recruited 281 pregnant women who regularly attended ACs, but only 188 who gave birth at our academic tertiary hospital were included in the final analysis. Socio-demographic characteristics, peripartum data (cervical dilatation at the time of admission to the hospital, duration of labor, and mode of delivery), and obstetric interventions (cesarean section (C/S) rate and rate of instrumental vaginal birth and episiotomy) were recorded, and the level of HL was assessed using the European Health Literacy Survey Questionnaire (HLS-EU-Q16). HL levels did not significantly affect peripartum and postpartum outcomes. **Results**: The study results showed that HL levels did not impact labor duration and newborn Apgar scores (*p* > 0.05). Patient education levels and employment status affected the peripartum duration of labor (*p* = 0.048 and *p* = 0.001, respectively). There were no differences in the HL total score and subscale scores (*p* > 0.05) between nulliparous and multiparous patients, and the rate of primary C/S was similar in both groups. **Conclusions**: HL levels did not impact delivery (peripartum) outcomes in pregnant women who attended antenatal classes. However, the primary C/S rate was similar between the nulliparous and multiparous groups, which may indicate that antenatal education services can correct the negative impact of low HL levels on the primary C/S rate.

## 1. Introduction

Health literacy (HL) is defined as the degree to which individuals have the “capacity to obtain, process, and understand basic health information and services needed to make appropriate health decisions” [1]. Nutbeam has identified HL as a measurable outcome of health education interventions [2]. Adults with low health literacy encounter barriers to health services and are less likely to understand medical advice [3]. Low HL is associated with many adverse health outcomes, improper interpretation of medication and nutrition, and decreased use of preventive services and educational activities [4].

It is essential for a woman to have appropriate knowledge about pregnancy and delivery in order to manage the decisions taken during childbirth. Antenatal classes (ACs) are educational services that provide pregnant women with knowledge of perinatal physiology and basic baby care skills, preparing expectant mothers for pregnancy and childbirth. Before ACs, information about birth and parenting was given more traditionally by people who had a previous birth experience in the family [5]. As a result of the increase in women’s education levels and the working female population, the process of obtaining information about the pregnancy and delivery process has been transferred from family members to ACs for the provision of less traditional, more professional support [6].

In ACs, pregnant women, especially those who will become mothers for the first time (nulliparous), are informed about the pregnancy and delivery process. A study examining factors associated with cesarean birth in nulliparous women has highlighted the need for clinicians to develop strategies to safely reduce the number of C/Ss in nulliparous women, which will ultimately lead to a reduction in the number of repeat C/Ss in multiparous women [7]. It is known that ACs play an essential role in women’s decisions about their health during pregnancy and breastfeeding [8,9]. Attendance of AC courses could positively influence the choice of delivery method and reduce the number of unnecessary C/Ss [10].

J.D. Stafford et al. noted that “little is known about the association of health literacy on the postpartum outcome” [11]. The purpose of modern technology and educational organizations in medicine is to make the assessment process easier for physicians and, in addition, to make it easier for patients to receive medical services. According to this goal, the present study aimed to examine and compare the impact of maternal health literacy levels on delivery outcomes between nulliparous and multiparous women who attended antenatal classes. Individuals with higher education levels or stable financial status tend to have better health literacy, which, in turn, can positively impact their healthcare outcomes [2,4]. Understanding how socio-demographic factors influence health literacy in antenatal class participants is essential for a comprehensive analysis of the primary objective—assessing the impact of maternal health literacy on delivery outcomes, considering the differences between nulliparous and multiparous women.

## 2. Materials and Methods

### 2.1. Study Population and Data Collection

The present study is a prospective observational study that included 281 native-language-speaking pregnant women who regularly attended antenatal classes, but only 188 who met the inclusion criteria were included in the final analysis between 1 January 2020 and 1 September 2020. The inclusion criteria were participants who could speak and understand their native language and were willing and able to provide informed consent. The exclusion criteria were conditions or diseases that prevented the patients from completing questionnaires: loss of vision, hearing, or sensory, motor, and cognitive (e.g., dementia, psychosis) skills. Of the original 281 patients, 93 were excluded from the study: 38 patients had comorbidities (gestational hypertension, gestational diabetes, overt diabetes), 21 patients had a poor obstetric history (recurrent pregnancy loss, early neonatal deaths, stillbirths, intrauterine fetal loss, intrauterine growth retardation, and congenital anomalies), and 7 patients had placental abnormalities. Since these conditions can affect maternal and fetal health, subsequently leading to an increase in the rate of obstetric indications for primary cesarean section, those patients were excluded from the trial. The remaining 27 patients who were not included in the study did not have accessible maternal, peripartum, or neonatal data. All participants were informed about the purpose of this study. Socio-demographic characteristics (SDCs) included age, education level, financial and employment status, and the number of family members living in the household.

Obstetrics data included patients’ gravida and parity, abortion history, and elective curettage (E/C) history. Peripartum data included cervical dilatation at the time of admission to the hospital, duration of labor, type of delivery, and obstetric interventions recorded for each patient in an active stage of labor. To avoid bias or interference in the physical examination, cervical dilation measurement was performed upon admission by two healthcare professionals: the midwife and the obstetrician in the emergency department. Obstetric interventions included cesarean section (C/S), instrumental vaginal birth (delivery), and episiotomy. The pregnant women were divided into nulliparous and multiparous groups, and HL, SDCs, perinatal data levels, and obstetric interventions were compared between these groups. The duration of labor is defined in this study as the period from patient admission to the hospital with active contractions until the delivery of the newborn. To assess the duration of labor, 41 patients from the multiparous C/S group were excluded from the analysis since these patients had indications for planned C/S, and the incidence of episiotomy was evaluated only in the vaginal delivery group. The primary outcome of this study was to identify the impact of maternal health literacy on delivery outcomes, focusing specifically on the differences in primary cesarean section (C/S) rates between nulliparous and multiparous women who attended antenatal classes. Additionally, secondary outcomes included examining the influence of socio-demographic factors and health literacy levels on delivery outcomes among antenatal class participants.

AC education is offered to all pregnant women, regardless of education and socioeconomic background, at the early stage of the second (II) trimester of pregnancy in our hospital. Every participant in the present study completed the prenatal education course, consisting of 5 sessions. The antenatal education courses, conducted by midwives under the supervision of an obstetrician, include video, educational, and theoretical parts. Each session lasts from one to one and a half hours. All training providers are qualified to provide training by the Ministry of Health and complete a course every 5 years to keep their qualifications up to date. In addition, all lessons are carried out under the supervision of a physiotherapist, dietician, and psychologist to ensure that the participants do not experience any physical or mental conflicts.

Homogeneous antenatal class education: All participants in this study received the same antenatal class (AC) education, which served to standardize the levels of information and support provided to pregnant women. This helped reduce the potential impact of varying socio-demographic backgrounds on the study’s outcomes, ensuring that the differences in delivery outcomes were not solely due to disparities in information access or knowledge.

The prenatal education courses in the present study were structured as follows:

1. Lesson: Safe Pregnancy Process;
2. Lesson: Methods of Coping with Birth Pain;
3. Lesson: Exercises During Pregnancy;
4. Lesson: Birth Process;
5. Lesson: Postpartum Period, Breastfeeding, Baby Care, and Family Planning Methods.

The socio-demographic characteristics, obstetrics data, and the European Health Literacy Survey Questionnaire (HLS-EU-Q16) (Table A1) scale were completed in the first lesson. A questionnaire was designed to collect information on HL and was carried out with face-to-face interviews between patients and clinicians (midwife, obstetrician, family physician). The HLS-EU-Q16 scale, consisting of 16 questions, was used to assess HL. The scale includes three subdomains: “Health Care (H.C.)”, “Disease Prevention (D.P.)”, and “Health Promotion (H.P.)” [12]. The first, second, and third subdomains include the 1st to 7th, 8th to 12th, and 13th to 16th questions, respectively. The scores to be obtained from the overall HLS-EU-Q16 scale and its subdomains vary between 0 and 50. The time taken to complete each questionnaire was ten to fifteen minutes. All participants provided informed consent before they participated in the study. This study was approved by the institution’s ethics committee (2011-KAEK-26/2019-21/12).

### 2.2. Statistical Analysis

A pilot study was conducted to determine the required sample size. Based on the results of the pilot study, a priori power analysis was performed. This study included 11 patients who met the inclusion and exclusion criteria and underwent cesarean section (C/S), as well as 12 patients with a normal vaginal delivery (NVD). Health literacy levels were assessed using the HLS-EU-Q16 scale, and the total scale scores were calculated for both groups. Patients who underwent a C/S had a mean score of 30.02 ± 9.79, while those with an NVD achieved a mean score of 36.11 ± 16.02. The effect size (Cohen’s d) was calculated as d = 0.46. Following this, a priori power analysis was performed assuming a type I error rate of 5%, an allocation ratio of 1, and a target power level of 85%. The noncentrality parameter was calculated as 3.02, and the critical t value was determined to be 1.97. Additionally, a potential 10% loss rate was accounted for, resulting in a final required sample size of 172 patients. Analyses were conducted using the G*Power software v3. The Kolmogorov–Smirnov test was used to assess whether the variables followed a normal distribution. Variables were reported as median (minimum–maximum) values. According to the normality test results, the Mann–Whitney U and Kruskal–Wallis tests were used for intergroup comparisons. The chi-square test was used to compare categorical variables. The Cronbach’s alpha coefficient was used to examine the internal consistency of the Health Literacy Scale. The reliability coefficients of the Health Literacy Scale and its subscales were found to be α = 0.75 for healthcare, α = 0.70 for disease prevention, α = 0.70 for health promotion, and α = 0.87 for the general scale. SPPS (IBM Corp. Released 2012. IBM SPSS Statistics for Windows, Version 21.0. Armonk, NY, USA: IBM Corp.) software was used for performing statistical analyses, and *p* < 0.05 was set as the threshold for statistical significance.

## 3. Results

In total, 188 pregnant women and 188 neonates were included in the study. The median age of the participants was 26 years (19–44 years). The demographic and obstetric characteristics of the participants are presented in Table 1. The total sample size was *n* = 188; however, some participants did not provide information on certain demographic variables, such as education level, employment status, and the number of family members, likely due to privacy concerns regarding their family structure. This accounts for the variations in the sample size for those specific variables.

The HL levels of the participants are shown in Table 2.

When the HL levels were examined, it was observed that more than 73.9% (139/188) of the participants had adequate (sufficient and excellent) HL levels according to the total and sub-dimension scores obtained from the HL scale.

The relationship between HL levels and socio-demographic characteristics is shown in Table 3.

There was no relationship between age and scores obtained from the HLS-EU-Q16 scale. The scores obtained from the general HL-EU-Q16 scale and the sub-dimensions of healthcare and disease prevention differed according to education level (*p* = 0.01, *p* = 0.03, and *p* = 0.01, respectively). In the subgroup analyses carried out for the overall scale score, the scale scores of the participants with education above high school level (39.58 vs. 34.38, *p* = 0.02) and the participants with a high-school-level education (38.54 vs. 34.38, *p* = 0.03) were higher than those of the participants with a lower education level. Based on the results of the subgroup analyses for the scale’s healthcare dimension, it was determined that the scale scores of the participants whose education was above high school level were higher than those of participants whose education was below high school level (39.29 vs. 35.71; *p* = 0.04). The subgroup analyses performed for the disease prevention dimension showed that the scale scores of the participants with a higher education level (40 vs. 31.67, *p* = 0.01) and the participants with a high-school-level education (40 vs. 31.67, *p* = 0.01) were higher than those of participants with a lower education level. The scores obtained from the health promotion sub-dimension did not differ by education level (*p* = 0.38). Overall, the healthcare and disease prevention scores were higher for employed patients (*p* = 0.02, *p* = 0.02, and *p* = 0.04), whereas there was no difference between employed and unemployed patients in terms of the health promotion score (*p* = 0.12). In addition, it was found that the reported health literacy scores did not differ according to financial status or number of family members (*p* > 0.05).

The relationship between HL and obstetric outcomes is reported in Table 4, and the relationship between socio-demographic characteristics and obstetric outcomes is reported in Table 5.

Table 4 shows no association between HL scores, education and financial status, and type of delivery and episiotomy (*p* > 0.05). There was no association between HL scores and labor duration, cervical dilatation, or APGAR scores (*p* > 0.05).

As shown in Table 5, the duration of labor differed according to education level (*p* = 0.05). Subgroup analyses showed that the labor duration of patients educated above a high school level was longer than that of patients educated below a high school level (*p* = 0.01). Cervical dilatation and APGAR scores did not differ according to educational level (*p* > 0.05). Labor duration did not differ according to financial status (*p* > 0.05). Cervical dilatation differed according to financial status, but no difference was observed between the groups in the subgroup analyses (*p* > 0.05). APGAR scores did not differ according to financial status (*p* > 0.05). The proportion of employees was higher in the episiotomy group than in the non-episiotomy group (*p* = 0.03). Labor duration was higher in employed patients (*p* = 0.01). Cervical dilatation measurements were higher in unemployed participants.

Table 6 shows that the age of multiparous patients was higher than that of nulliparous patients (26 years vs. 28 years; *p* < 0.001). There was a difference between nulliparous and multiparous patients in terms of education level (*p* < 0.001). Among multiparous patients, the proportion of patients with below high school and high school education levels was higher (35.20% vs. 13.90%, *p* < 0.05, and 42.90% vs. 24.10%, *p* < 0.05, respectively) than among nulliparous patients, whereas the proportion of patients educated above a high school level was higher in the nulliparous patient group (62% vs. 21.90%; *p* < 0.05). Financial status did not differ between the groups (*p* = 0.681). There was a difference between nulliparous and multiparous patients in terms of employment status (*p* < 0.001). The proportion of employed patients was higher in the nulliparous patient group (72.50% vs. 39.20%). There was no difference between groups in terms of family members (*p* = 0.250). According to the overall HL scale and the scores obtained from the subscales, there was no difference between nulliparous and multiparous patients. In the multiparous group, cervical dilatation was greater at admission (3 cm vs. 4 cm; *p* = 0.01). It was observed that there was no difference between the groups according to the type of delivery (*p* = 0.281). The duration of labor was higher in the nulliparous group than in the multiparous group (6 h vs. 4 h; *p* = 0.002). The incidence of episiotomy was higher in the nulliparous group (62.5%, 50/80 vs. 39.8%, 43/108; *p* = 0.01).

## 4. Discussion

HL is the degree of ability to understand health-related information, promote health, and prevent disease; it is one of the most essential factors that improve communication between healthcare providers and patients in healthcare systems [13]. The levels of HL are essential not only for patients but also for service providers. A study published in 2021, which the authors declared to be the first of its kind, examined health literacy among physicians. Mor-Anavy et al. revealed that higher HL among providers is associated with more favorable attitudes toward health literacy promotion [14]. The authors also reported a significant association between the attitudes toward health literacy promotion and the use of communication methods. In order to avoid those negative impacts on pregnant women’s education, in our study, the AC training was carried out by a multidisciplinary team and with the participation of at least two healthcare providers. In addition, all patients with comorbidities or a poor obstetric history were excluded from the study, as these patients require an individualized approach.

The significance of health literacy was underscored anew during the COVID-19 pandemic, highlighting its critical importance [15]. Our country’s general health literacy index was 30.4%, with this study revealing that 24.5% of individuals had inadequate and 40.1% had problematic levels of health literacy, indicating significant challenges in public understanding and management of health information [16]. The level of education is one of the most significant indicators affecting HL [17]. As we know, a low level of education is associated with low levels of HL. The results of the present study likewise emphasized the association between the level of HL and patients’ educational status. Our study showed that patients with low education levels have low levels of HL. Various studies on HL have shown that the main factors that lead to insufficient HL are age, gender, education, and financial status [18,19]. However, our study results showed no association of HL scores with patients’ age, financial status, or number of family members. In our study, the participants’ employment status was the most significant factor influencing their HL. Hakkak et al. found that 98.2% of pregnant women had optimal health literacy [20]. In contrast, another study from Turkiye showed that only 7.5% of women of reproductive age had adequate or excellent health literacy [21]. In contrast to the findings of Mahsun and Demirci, the results of the present study showed that overall, HL was sufficient in 41.50% and excellent in 32.40% of participants. This may be due to the fact that our study population consisted entirely of pregnant women (aged 19–44 years) participating in the AC.

The National Institute for Health and Care Excellence (NICE) published a review (2021) that researched the effectiveness of ACs and groups in preparing pregnant women for labor [22]. The NICE guidelines suggest that antenatal education may help women present for labor with greater cervical dilation. The committee pointed out that a more dilated cervix on presentation to labor can reduce the need for interventions at the hospital. Analyzing the correlation between HL and different SDCs in pregnant women attending ACs may provide important context for interpreting the results of our primary research question, as differences in HL based on socio-demographic factors may explain differences in delivery outcomes. The present study revealed that the participants educated to high school level and above, as well as those with a profession, had less cervical dilatation at hospitalization. A less dilated cervix at admission affects the duration of labor, which is also longer in patients with high education levels. A review focused on investigating the effectiveness of assessment of early-stage labor versus immediate hospitalization found that assessment and support interventions during early labor may reduce epidural anesthesia and labor augmentation while increasing maternal satisfaction with giving birth [23]. This may explain why women with higher education levels and those with professions had a longer labor duration in our study. The highly educated population of patients was more aware of obstetric emergencies (E.O.), as detailed in the fourth lesson of the antenatal education classes. This population of patients applied to the hospital at the early stage of labor for professional assessment by obstetricians or midwives.

Bello et al. examined the relationship between maternal health literacy and the utilization of maternal healthcare services, noting that women with inadequate HL are less likely to engage with healthcare providers, and approximately half of the study participants had poor pregnancy outcomes. Their findings suggest that health literacy plays a critical role in the decision-making process regarding pregnancy outcomes [24]. This further supports the claim that health literacy influences the choices that pregnant women, especially nulliparous women, make during childbirth. The NICE guidelines emphasize the necessity for all nulliparous women to have the opportunity to receive adequate information about pregnancy and the birth process [22]. Nulliparous women experiencing childbirth for the first time may be more influenced by their health literacy levels, which could play a role in encouraging vaginal delivery [25]. Despite the absence of any difference between nulliparous and multiparous patients according to the HL scale, our data show that the vaginal delivery rates did not differ, and the rate of a primary C/S was not increased in the nulliparous group. A review aiming to investigate the reasons behind maternal requests for elective cesarean section without maternal or fetal indication concluded that “Proposals for comprehensive health promotion programs and interventions should be made to reduce unnecessary cesarean section and improve the performance of vaginal delivery” [26]. Nulliparous and multiparous patients had the same levels of HL in our study, but it should not be forgotten that all participants received professional knowledge about the birth process in the ACs (4. Lesson: Birth Process). In our study, the fact that the primary C/S rate did not show any increase in nulliparous patients and was similar to that of multiparous patients can be explained by the fact that educational services such as ACs can equalize HL levels and improve the peripartum and postpartum outcomes of pregnant women.

Numerous studies agree that women who attend ACs are at a lower risk of cesarean section [27,28,29]. According to an ACOG opinion published in 2014, modifiable factors, such as patient preferences and practice variation among hospitals, systems, and healthcare providers, are increasing the preference for cesarean delivery [30]. Many expectant mothers are anxious about trauma to the newborn when passing through the vaginal canal. A study found that the main reason for attending prenatal classes for pregnant women was to reduce their anxiety about childbirth and the birth process [31,32]. This is how pregnant women without appropriate antenatal education explained their baseless requests for elective C/S. A study conducted by Ricchi et al. with 147 pregnant women showed that providing antenatal preparation courses as psychological support for prospective mothers could reduce the rate of interventions during labor (e.g., frequency of epidural analgesia) but did not impact the rate of C/S [33]. To avoid the confounding effect for comparison of the outcomes (primary cesarean section rate, duration of labor, episiotomy rate) between nulliparous and multiparous pregnancies, 41 planned (elective) C/S patients were excluded from the analysis. Our study showed that nulliparous patients, compared with the group with more than one birth experience, despite having the same levels of HL, did not have a higher rate of C/S. After multiparous patients with a previous C/S history (planned C/S) were eliminated, no difference was found in the primary C/S rate between nulliparous and multiparous patients. As we know, multiparous patients have experience of childbirth, but nulliparous patients are more vulnerable due to their lack of experience. No difference in C/S rate between the two groups in the absence of a difference in HL level may be an indicator of the success of antenatal education. This outcome of our study reminds us of the Latin expression “Gutta cavat lapidem”, which means “A water drop hollows a stone”. Despite the lack of any difference in the levels of HL between multiparous and nulliparous patients, the similar levels of C/S led to the conclusion that the proper use of ACs can reduce the incidence of primary C/S in nulliparous pregnancies. On the other hand, our study results showed that age, education, and being employment were higher in nulliparous than in multiparous participants. Recent studies reported that HL is lower in the elderly population, in individuals with low education and income levels, and in those who cannot use their native language [4]. Low HL levels cause difficulties in understanding health information and fulfilling processes and instructions, as well as problems related to the ineffective use of health services [4,34,35]. Higher levels of education are a fundamental condition that can improve the HL of pregnant women, leading to easy understanding and improvisation of health-related information in ACs. However, the rate of interventions between the nulliparous and multiparous groups after antenatal classes showed a difference in our study. After the elimination of the confounding factors, the results of the present study show that the use of ACs did not affect the frequency of episiotomies, which was higher in the nulliparous group. Additionally, our study did not observe neonatal obstetric complications in any of the 188 newborns, all of whom had high Apgar scores.

### Limitations

Before concluding, we would like to touch on this study’s limitations. As a rule, the limitations of a study become apparent during the process of its completion. The discovery of limitations is important because their identification plays a huge role in planning new research on this topic. Recent research aimed to explore the influence of healthcare providers’ unconscious or implicit biases on patient treatment outcomes. Bias is a lack of objectivity combined with a preference for a particular person, group, or item [36]. This attitude could potentially impact patient outcomes. Since the training in the present study was carried out by the same qualified team authorized by the Ministry of Health and was conducted in the same location, the impact of the provider on the cohort was not analyzed in our study. Another limitation is that our study was designed for the antenatal-educated population; therefore, patients who did not attend ACs were not included. A study designed with a control group may possibly provide a more definitive conclusion.

## 5. Conclusions

This study did not find a significant relationship between health literacy levels and delivery outcomes, but the rate of primary C/S was similar between nulliparous and multiparous women who attended antenatal classes. Despite the similar HL levels in both groups, the lack of increase in primary C/S rate in nulliparous women may be explained by the effectiveness of AC educational services. These findings suggest that while AC education is beneficial for increasing knowledge and preparedness for childbirth, its impact on mitigating the effects of low health literacy on obstetric outcomes remains inconclusive. Future studies with a control group will be necessary to determine whether antenatal education can effectively improve health literacy and, in turn, influence delivery outcomes.

## Figures and Tables

**Table 1:** Demographic and obstetric characteristics of the participants.

Age (years) (*n* = 188)	26 (19–44) (27.18 ± 4.38)
BMI (kg/m^2^)	29.05 (20.69–44.63)
Smoker (*n* = 188)	13 (6.9%)
Chronic Disease (*n* = 188)	8 (4.3%)
Education Level (*n* = 184)	
Below high school	48 (26.1%)
High school	64 (34%)
Above high school	72 (38.3%)
Financial Status (*n* = 180)	
Low	24 (13.3%)
Moderate	126 (7%)
Good	30 (16.7%)
Profession (*n* = 148)	
Employed	81 (54.7%)
Unemployed	67 (45.3%)
Family Member (*n* = 73)	
With mother-in-law	25 (34.2%)
Compact family	48 (65.8%)
Planned Pregnancy (*n* =171)	
Yes	131 (76.6%)
No	40 (21.3%)
Gravida (*n* = 188)	2 (0–5)
Parity (*n* = 188)	1 (0–3)
Abortion (*n* = 188)	0 (0–5)
Curettage History (*n* = 188)	
Absent	104 (55.3%)
One	25 (13.3%)
More than one	59 (31.4%)

Data are presented as the median (minimum–maximum), (mean ± st. deviation), and *n* (%).

**Table 2:** The health literacy levels of the participants.

Health Literacy	Inadequate (0–25)	Problematic (>25–33)	Sufficient (>33–42)	Excellent (>42–50)
General	14 (7.4%)	35 (18.6%)	78 (41.5%)	61 (32.4%)
Healthcare	11 (5.9%)	25 (13.3%)	85 (45.2%)	67 (35.6%)
Disease Prevention	29 (15.4%)	25 (13.3%)	69 (36.7%)	65 (34.6%)
Health Promotion	21 (11.2%)	14 (7.4%)	81 (43.1%)	72 (38.3%)

Data are presented as *n* (%).

**Table 3:** The relationship between health literacy levels and socio-demographic characteristics.

		Overall HL	Health Care	Disease Prevention	Health Promotion
Age (*n* = 188)	r_s_	−0.02	−0.06	0.01	0.06
	*p*-value	0.98	0.39	0.88	0.39
Education					
Below high school (*n* = 48)		34.38 (9.38–50)	35.71 (7.14–50)	31.67 (3.33–50)	37.50 (8.33–50)
High school (*n* = 64)		38.54 (21.88–50)	38.09 (19.05–50)	40 (13.33–50)	37.50 (25–50)
Above high school (*n* = 72)		39.58 (21.88–50)	39.29 (21.43–50)	40 (13.33–50)	37.50 (8.33–50)
*p*-value ^a^		0.01	0.03	<0.01	0.38
Financial Status					
Low (*n* = 24)		34.90 (20.83–50)	36.90 (21.43–50)	31.67 (13.33–50)	35.42 (8.33–50)
Moderate (*n* = 126)		38.54 (17.71–50)	38.09 (9.52–50)	36.67 (3.33–50)	37.50 (8.33–50)
Good (*n* = 30)		40.10 (9.38–50)	38.09 (7.14–50)	40 (3.33–50)	41.67 (8.33–50)
*p*-value ^a^		0.26	0.37	0.22	0.42
Profession					
Employed (*n* = 81)		39.59 (18.75–50)	40.48 (21.43–50)	40 (3.33–50)	41.67 (8.33–50)
Unemployed (*n* = 67)		36.46 (9.38–50)	38.10 (7.14–50)	36.67 (3.33–50)	37.50 (8.33–50)
*p*-value ^b^		0.02	0.02	0.04	0.12
Family Member					
With mother-in-law (*n* = 25)		35.42 (21.88–50)	38.10 (21.43–50)	33.33 (13.33–50)	33.33 (20.83–50)
Compact family (*n* = 48)		37.50 (17.71–48.96)	38.10 (19.05–50)	40 (3.33–50)	39.58 (20.83–50)
*p*-value ^b^		0.28	0.86	0.10	0.18

Data are presented as the median (minimum–maximum). HL: health literacy. ^a^: Kruskal–Wallis test, ^b^: Mann–Whitney U test, r_s_: Spearman correlation coefficient.

**Table 4:** The relationship between health literacy levels and obstetric outcomes.

	Type of Delivery			Episiotomy			Labor Duration (Hr)		Cervical Dilatation (cm)		APGAR I		APGAR V	
	C/S (*n* = 87)	Vaginal Delivery (*n* = 101)	*p*-Value	Present (*n* = 93)	Absent (*n* = 95)	*p*-Value								
Health Literacy														
Overall	37.50 (14.58–50)	38.54 (9.38–50)	0.21 ^b^	39.58 (9.38–50)	37.50 (14.58–50)	0.10 ^b^	r_s_	0.01	r_s_	−0.02	r_s_	0.03	r_s_	0.02
							*p*	0.94	*p*	0.85	*p*	0.70	*p*	0.77
Health Care	38.10 (9.52–50)	38.10 (7.14–50)	0.39 ^b^	40.48 (7.14–50)	35.71 (9.52–50)	0.17 ^b^	r_s_	0.07	r_s_	−0.11	r_s_	−0.08	r_s_	−0.03
							*p*	0.33	*p*	0.26	*p*	0.30	*p*	0.67
Disease Prevention	33.33 (3.33–50)	36.67 (3.33–50)	0.17 ^b^	36.67 (3.33–50)	33.33 (3.33–50)	0.12 ^b^	r_s_	0.01	r_s_	0.07	r_s_	0.07	r_s_	0.04
							*p*	0.89	*p*	0.95	*p*	0.37	*p*	0.61
Health Promotion	37.50 (8.33–50)	41.67 (8.33–50)	0.50 ^b^	41.67 (8.33–50)	37.50 (8.33–50)	0.32 ^b^	r_s_	−0.08	r_s_	0.10	r_s_	0.11	r_s_	0.07
							*p*	0.28	*p*	0.30	*p*	0.13	*p*	0.37

Data are presented as the median (minimum–maximum) and *n*%. HL: health literacy. ^b^: Mann–Whitney U test, r_s_: Spearman correlation coefficient.

**Table 5:** The relationship between social factors and obstetric outcomes.

	Type of Delivery			Episiotomy			Labor Duration (Hr)		Cervical Dilatation (cm)		APGAR I		APGAR V	
	C/S (*n* = 87)	Vaginal Delivery (*n* = 101)	*p*-Value	Present (*n* = 93)	Absent (*n* = 95)	*p*-Value								
Education														
Below high school	25 (29.4%)	23 (23.2%)	0.27 ^c^	21 (22.8%)	27 (29.3%)	0.20 ^c^	3 (1–36)	*p* = 0.05 ^a^	4 (1–9)	*p* = 0.16 ^a^	9 (9–9)	*p* = 0.48 ^a^	10 (10–10)	*p* = 0.51 ^a^
High school	32 (37.6%)	32 (32.3%)		29 (31.5%)	35 (38%)		5 (1–36)		3 (1–9)		9 (7–9)		10 (8–10)	
Above high school	28 (32.9%)	44 (44.4%)		42 (45.7%)	30 (32.6%)		6 (0–30)		3 (1–8)		9 (5–9)		10 (8–10)	
Financial Status														
Low	6 (7.2%)	18 (18.6%)	0.06 ^c^	16 (18%)	8 (8.8%)	0.14 ^c^	5.50 (1–36)	*p* = 0.64 ^a^	4 (2–9)	*p* = 0.02 ^a^	9 (9–9)	*p* = 0.16 ^a^	10 (10–10)	*p* = 0.06 ^a^
Moderate	64 (77.109	62 (63.9%)		57 (64%)	69 (75.8%)		4 (1–30)		3 (1–8)		9 (7–9)		10 (8–10)	
Good	13 (15.7%)	17 (17.5%)		16 (18%)	14 (15.4%)		3 (0–30)		4 (2–9)		9 (5–9)		10 (8–10)	
Profession														
Employed	31 (47%)	50 (61%)	0.089 ^c^	48 (63.2%)	33 (45.8%)	0.03 ^c^	6 (0–36)	*p* = 0.01 ^b^	3 (1–9)	*p* = 0.01 ^b^	9 (5–9)	*p* = 0.882 ^b^	10 (8–10)	*p* = 0.66 ^b^
Unemployed	35 (53%)	32 (39%)		28 (36.8%)	39 (54.2%)		3 (1–36)		4 (1–9)		9 (7–9)		10 (8–10)	

Data are presented as the median (minimum–maximum) and *n*%. HL: health literacy. ^a^: Kruskal–Wallis test, ^b^: Mann–Whitney U test, ^c^: chi-square test, r_s_: Spearman correlation coefficient.

**Table 6:** Comparison of the HL levels, socio-demographic characteristics, and peripartum and postpartum results of nulliparous and multiparous women.

	Nulliparous (*n* = 80)	Multiparous (*n* = 108)	*p*-Value
Age (years)	26 (20–36)	28 (19–44)	<0.001 ^a^
Education			
Below high school (*n* = 48)	11 (13.9%)	37 (35.2%)	<0.001 ^c^
High school (*n* = 64)	19 (24.1%)	45 (42.9%)	
Above high school (*n* = 72)	49 (62%)	23 (21.9%)	
Financial Status			
Low (*n* = 24)	10 (13%)	14 (13.6%)	0.681 ^c^
Moderate (*n* = 126)	52 (67.5%)	74 (71.8%)	
Good (*n* = 30)	15 (19.5%)	15 (14.6%)	
Profession			
Employed (*n* = 81)	50 (72.5%)	31 (39.2%)	<0.001 ^c^
Unemployed (*n* = 67)	19 (27.5%)	48 (60.8%)	
Family Member			
With mother-in-law (*n* = 25)	10 (27.8%)	15 (40.5%)	0.250 ^c^
Compact family (*n* = 48)	26 (72.2%)	22 (59.5%)	
Health Literacy (HL)			
Overall HL	38.54 (21.88–50)	37.50 (9.38–50)	0.063 ^b^
Health care	39.29 (21.43–50)	38.10 (7.14–50)	0.440 ^b^
Disease prevention	36.67 (13.33–50)	36.67 (3.33–50)	0.910 ^b^
Health promotion	37.50 (8.33–50)	37.50 (8.33–50)	0.258 ^b^
Cervical Dilatation (cm)	3 (1–8)	4 (1–9)	0.001 ^a^
Type of Delivery			
C/S (*n* = 46 *)	28 (35%)	18 (26.9%)	0.289 ^c^
Vaginal delivery (*n* = 101)	52 (65%)	49 (73.1%)	
Duration of Labor *	6 (0–36)	4 (1–36)	0.002 ^a^
Episiotomy **	50 (62.5%)	43 (39.8%)	0.002 ^c^

Data are presented as the median (minimum–maximum) and *n*%. HL: health literacy. * A total of 41 patients were excluded from the multiparous C/S group due to having had a previous (planned) C/S. ** For 101 vaginal deliveries, 2 nulliparous and 6 multiparous patients had no episiotomy. ^a^: Mann–Whitney U test, ^b^: Kruskal–Wallis test, ^c^: Chi-square test.

## Data Availability

The data presented in this study are available from the corresponding author upon request.

## Appendix A

**Table A1:** HLS-EU-Q16 survey questionnaire.

	Very Difficult	Fairly Difficult	Fairly Easy	Very Easy
1. Find information on treatments of illnesses that concern you?				
2. Find out where to get professional help when you are ill?				
3. Understand what your doctor says to you?				
4. Understand your doctor’s or pharmacist’s instruction on how to take a prescribed medicine?				
5. Judge when you may need to get a second opinion from another doctor?				
6. Use information the doctor gives you to make decisions about your illness?				
7. Follow instructions from your doctor or pharmacist?				
8. Find information on how to manage mental health problems like stress or depression?				
9. Understand health warnings about behavior such as smoking, low physical activity and drinking too much?				
10. Understand why you need health screenings?				
11. Judge if the information on health risks in the media is reliable?				
12. Decide how you can protect yourself from illness based on information in the media?				
13. Find out about activities that are good for your mental well-being?				
14. Understand advice on health from family members or friends?				
15. Understand information in the media on how to get healthier?				
16. Judge which everyday behavior is related to your health?				
